# The effect of team collaboration and continuity of care on health and disability among rehabilitation patients: a longitudinal survey-based study from western Norway

**DOI:** 10.1007/s11136-019-02216-7

**Published:** 2019-05-29

**Authors:** Merethe Hustoft, Eva Biringer, Sturla Gjesdal, Vegard Pihl Moen, Jörg Aβmus, Øystein Hetlevik

**Affiliations:** 1 Centre for Habilitation and Rehabilitation in Western Norway, Haukeland University Hospital, Bergen, Norway; 2 Department of Global Health and Primary Health Care, University of Bergen, Bergen, Norway; 3 Section of Research and Innovation, Helse Fonna Local Health Authority, Haugesund/Stord, Norway; 4 Centre for Clinical Research, Haukeland University Hospital, Bergen, Norway

**Keywords:** Continuity of patient care, Rehabilitation, Disability evaluation, Interprofessional relations, Patient-rated outcome measures, Relational coordination

## Abstract

**Purpose:**

The purpose of this study was to investigate how changes in patient-rated health and disability from baseline to after rehabilitation were associated with communication and relationships in rehabilitation teams and patient-rated continuity of care.

**Methods:**

Linear models were used to assess the associations between relational coordination [RC] and Nijmegen Continuity Questionnaire-Norwegian version [NCQ-N] with changes in the World Health Association Disability Assessment Schedule 2.0 [WHODAS 2.0] and EuroQol EQ-VAS [EQ-VAS]. To express change in WHODAS 2.0 and EQ-VAS, the model was adjusted for WHODAS 2.0 and EQ-VAS baseline scores. Analyses for possible slopes for the various diagnosis groups were performed.

**Results:**

A sample of 701 patients were included in the patient cohort, followed from before rehabilitation to 1 year after a rehabilitation stay involving treatment by 15 different interprofessional teams. The analyses revealed associations between continuity of care and changes in patient-rated health, measured with EQ-VAS (all *p* values < 0.01). RC communication was associated with more improvement in functioning in neoplasms patient group, compared to improvement of health among included patient groups. The results revealed no associations between NCQ-N and WHODAS 2.0 global score, or between RC in the rehabilitation teams treating the patients and changes in WHODAS 2.0 global score.

**Conclusion:**

The current results revealed that better personal, team and cross-boundary continuity of rehabilitation care was associated with better patient health after rehabilitation at 1-year follow-up. Measures of patient experiences with different types of continuity of care may provide a promising indicator of the quality of rehabilitation care.

**Electronic supplementary material:**

The online version of this article (10.1007/s11136-019-02216-7) contains supplementary material, which is available to authorized users.

## Background

Rehabilitation is considered one of the most important processes enabling attainment and maintenance of physical, mental, social and vocational activities for people with various health conditions and disabilities [1]. Somatic rehabilitation emphasises health and functioning through a continuous and coordinated process that extends over a period of time with a collaborating interprofessional rehabilitation team [2]. Self-rated health and disability have received increased attention in recent decades as important outcomes in rehabilitation [3].

While undergoing rehabilitation, patients are treated by an array of health care professionals in a team, not only during their stay in a rehabilitation centre, but also across multiple specialities and in different health care settings [4]. According to Donabedian’s health care quality model, high-quality structures of care should lead to improvements in clinical processes and subsequently improve patient outcomes [5]. Collaboration and coordination in interprofessional rehabilitation teams are important for ensuring good quality continuity of care and outcomes for patients [6–8]. Relational coordination (RC) among interprofessional team members has been found to improve patient outcomes [9] and impact care coordination [10].

Continuity of rehabilitation care occurs when patient experiences are linked to care over time or when the care is connected [11]. Continuity of care is considered to be essential for high-quality patient care [12–14] and is commonly framed as being composed of relational continuity (relationship between a patient and a provider over time), information continuity (availability and use of data from prior events during current patient encounters) and management continuity (coherent delivery of care from different health care professionals) [11, 13]. It is generally preferable for continuity of care to be measured from the patients’ perspective [15].

A large number of studies of continuity of care have examined the personal continuity between patients and general practitioner (GP) or health care professional delivering care over time and have typically been performed in primary health care settings [16, 17]. Few studies have investigated continuity of care in somatic specialised health care and even fewer have examined somatic rehabilitation settings [18–20]. Investigations of patients’ perceived personal, team and cross-boundary continuity in rehabilitation services are scarce [21]. In a recent study, we found associations between RC functions in interprofessional rehabilitation teams and the patient-rated continuity of care at 1-year follow-up [18]. Further, this previous study also indicated weak associations between RC subscale scores and patient-rated benefit in more general terms, most pronounced related to activities in daily living [18].

To the best of our knowledge, no previous studies have investigated the associations between interprofessional team functioning and continuity of care with changes in patient-rated health and disability longitudinally. Therefore, we assessed associations between RC in interprofessional rehabilitation teams and patient-rated continuity of care with changes in patient-rated health and disability.

### Aims

The current study sought to investigate how changes in patient-rated health and disability from baseline to after rehabilitation were associated with communication and relationships in rehabilitation teams and patient-rated continuity of care.

## Methods

### Study design

**Fig. 1:**
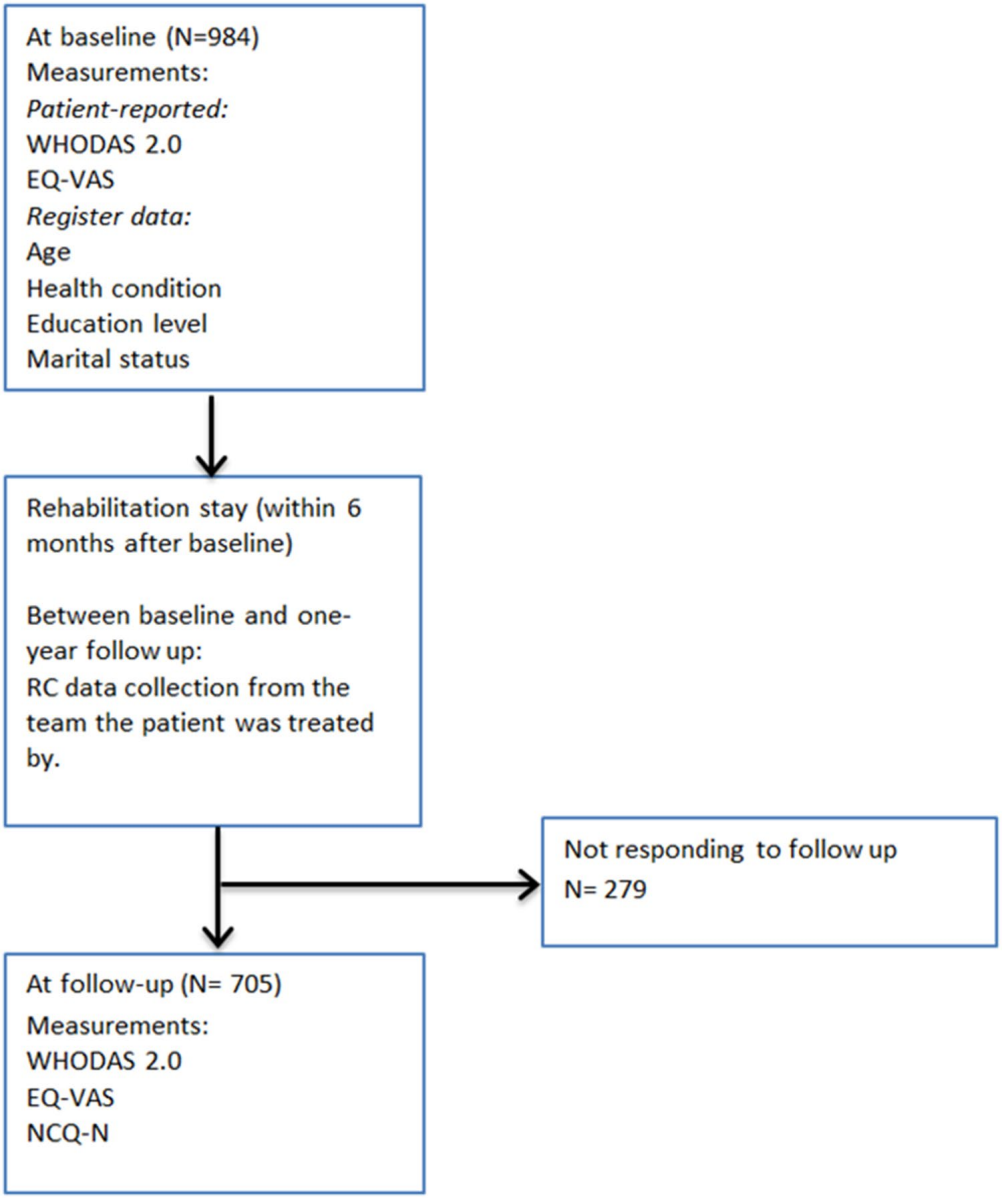
Flow chart for data collection

This study used a longitudinal survey-based design following a cohort of patients accepted for a rehabilitation stay in secondary health care services (Fig. 1). Survey data was collected when patients were recruited (baseline) and in a follow-up survey 1 year after baseline data collection. In between the two surveys, the patients had a rehabilitation stay in one of the centres. All patients included have taken part in a 3-week rehabilitation process treated by an interprofessional team comprising of a physician, occupational therapist, physical therapist, nurse and other relevant team members. Each of the seven rehabilitation centres in Western Norway provides interventions appropriate for the diagnostic group referred to the centre. As we aimed to include a large cohort of rehabilitation patients in Western Norway all patients who were referred with various diagnosis were included, and therefore a single specific intervention is not studied. RC in interprofessional teams were estimated by a survey among the professionals working in the rehabilitation centres.

### Participants

#### Patient cohort

All patients aged 18 and above who were accepted for rehabilitation in a rehabilitation centre in Western Norway between January 2015 and June 2015 were invited to participate (*n* = 2863). For baseline data collection, a total of 984 (34%) patients accepted the invitation to participate and provided written consent and a completed questionnaire. A 1-year follow-up questionnaire was sent to all participating patients (*n* = 984), and 705 patients (25% of the patient group invited at baseline) returned the questionnaire. We extracted 279 of the baseline participants from the analyses, as they did not respond to the 1-year follow-up survey. Four respondents were omitted from the analyses due to missing data on outcome variables. Finally, 701 (24% of the patient group invited at baseline) patients were included in the analyses (Table 1). Each patient respondent was linked to their corresponding interprofessional team from whom they received rehabilitation services during their stay in the rehabilitation centre. Further descriptions of the recruitment and inclusion process of patients and health care professionals have been reported in previous studies [18, 22, 23].

**Table 1:** Characteristics of included rehabilitation patients (*N* = 701) answering both baseline and the 1-year follow-up survey, and non-responders of the 1-year follow-up survey (*N* = 279)

Patient characteristics	Included patients (*N* = 701)	Non-responders at 1-year follow-up (*N* = 279)
Age mean (SD)		
Male	63 (13.4)	56 (12.83)
Female	60 (13.5)	52 (15.12)
Age group *n* (%)		
18–29	10 (1.4)	12 (4.6)
30–39	35 (5.0)	40 (14.5)
40–49	113 (16.1)	68 (24.5)
50–59	165 (23.5)	65 (23.5)
60–69	198 (28.3)	52 (18.8)
> 70	180 (25.7)	39 (14.1)
Missing	0 (0)	0 (0)
Sex *n* (%)		
Male	269 (38.0)	88 (31.5)
Female	432 (62.0)	191 (68.5)
Missing	0 (0)	0 (0)
Health conditions *n* (%)		
Neoplasms	49 (7.0)	16 (5.7)
Diseases in the nervous system	81 (11.6)	21 (7.5)
Diseases in the musculoskeletal system	356 (50.8)	130 (46.6)
Diseases in the circulatory system	60 (8.6)	48 (17.2)
Others^a^	152 (21.7)	64 (23.0)
Missing	3 (0.4)	0 (0)
Education level *n* (%)		
Elementary school	152 (21.7)	76 (27.2)
High school	328 (46.8)	128 (45.9)
College/University	213 (30.4)	67 (24.0)
Missing	8 (1.1)	8 (2.9)
Marital status *n* (%)		
Married	356 (50.8)	130 (46.6)
Unmarried, not divorced	189 (27.0)	83 (29.7)
Divorced	150 (21.4)	64 (22.9)
Missing	6 (0.9)	2 (0.7)

^a^Other health conditions included the following: endocrine, nutritional and metabolic diseases (*n* = 36); respiratory diseases (*n* = 35); diseases of the skin and subcutaneous tissue (23); injuries and external causes (*n* = 18); factors influencing self-rated health and contact with services (*n* = 7); mental and behavioural disorders (*n* = 12); symptoms, sign and abnormal clinical and laboratory findings, not elsewhere classified (*n* = 4); codes for special purposes (*n* = 6); diseases of the digestive system (*n* = 5); diseases of the blood and blood-forming organs, and certain disorders involving the immune mechanism (*n* = 1); diseases of the ear and the mastoid process (*n* = 1); diseases of the genitourinary system (*n* = 1); congenital malfunctions, and chromosomal abnormalities (*n* = 1); and certain infectious and parasitic diseases (*n* = 2)

#### Dependent variables and measurements

The World Health Organization Disability Assessment Schedule version 2.0 (WHODAS 2.0) was developed to correspond directly to the “activity and participation” dimension of the International Classification of Disability, Function and Health (ICF) [24] and has previously been used to evaluate disability in a generic rehabilitation group [25, 26]. WHODAS 2.0 is an extensively validated and used patient-rated generic self-evaluation survey instrument [22, 27, 28]. WHODAS 2.0 is translated into several languages, including Norwegian [22], and has been used in various health care settings, such as chronic care [24], stroke [29] and secondary rehabilitation services [23].

WHODAS 2.0 measures health and disability using 36 items across six domains [26] (number of items and Cronbach’s alpha from Norwegian validation study [22] in parentheses): cognition (six items, *α* = 0.87), mobility (five items, *α* = 0.85), self-care (four items, *α* = 0.77), getting along (five items, *α* = 0.75), life activities (eight items, *α* = 0.91) and participation (eight items, *α* = 0.83). Four items in the domain of *life activities* relate to the household and four items relate to work/study. Responses were given on a five-point Likert scale (one = none, two = mild, three = moderate, four = severe and five = extreme or cannot do). Scores were computed for each domain by adding the item responses representing each domain. Each domain score was transformed into a range from zero (best = no disability) to 100 (worst = full disability). A global score was calculated using either all 36 items or 32 items in cases where the four items regarding work/school were omitted because they did not apply to the participating patients [30]. The global score ranged from zero (best = no disability) to 100 (worst = full disability). The range scores for the domain and global scores were assessed as 0–4: no functional problem; 5–24: mild functional problem; 25–40: moderate functional problem; 50–95: severe functional problem and 95–100: total functional loss. The calculation of the WHODAS 2.0 domain and global scores was conducted according to the WHODAS 2.0 manual with complex scoring [26].

The EuroQol-5 dimension descriptive system (EQ-5D) includes a visual analogue scale (EQ-VAS) for measuring respondents’ overall health status [31–34]. The EQ-5D is an extensively validated and reliable generic health-related measurement tool [35–37], including validation in rehabilitation settings [31, 32]. EQ-5D has, among others, been used in primary care [38], geriatric health [39] and in somatic and community-based rehabilitation settings [23, 40]. EQ-5D is ideally used by self-evaluation [34]. Respondents indicated their self-rated health on a vertical, calibrated, line ranging from zero (“worst imaginable health state”) to 100 (“best imaginable health state”) [34].

#### Independent variables and measurements

The main independent variables in this study were the team-reported RC subscale scores and the patient-rated Nijmegen Continuity Questionnaire, Norwegian version, (NCQ-N) subscale scores.

RC is a self-reporting validated survey measuring team functions among members of interprofessional teams [20, 41]. The RC survey has recently been translated into Norwegian and validated within teams in specialised health care settings [42]. This study found a satisfactory two-factor solution (Cronbach’s alpha in parentheses); RC communication = four items: frequency, accuracy, timeliness and problem-solving (*α* = 0.93), RC relationship = three items: shared knowledge, shared goals, mutual respect) (*α* = 0.80) [42, 43]. Each item represents a question (e.g. “How *frequently* do members of the interprofessional team communicate with you about the rehabilitation patient?”). Responses were reported on a 5-point Likert scale (one = never, two = rarely, three = occasionally, four = often and five = always). RC has been used in various health care settings, such as primary health [44], hospital settings [42, 45] and secondary rehabilitation services [18]. RC subscale scores were obtained for all teams (*N* = 15) in all rehabilitation centres by conducting a survey among health care professionals (*N* = 124, 52% response rate). The RC subscale scores are reported as clustered mean scores for each team in this study, and scores were assigned to the patients treated by the respective teams.

The Nijmegen Continuity Questionnaire (NCQ) is a validated generic survey measuring continuity of care from the perspectives of the patients and consists of 28 items divided into six subscales [46, 47]. The NCQ has been used in primary care [19], chronic illness [48] and somatic rehabilitation [18, 49]. The NCQ has recently been translated into Norwegian (NCQ-N) [49]. In this study, we used two subscales of the NCQ-N for personal continuity (number of items and Cronbach’s alpha in parentheses): most important health care professional in the interprofessional rehabilitation team knows me (five items, *α* = 0.92), most important health care professional in the interprofessional rehabilitation team shows commitment (three items, *α* = 0.88) together with subscales regarding team continuity: collaboration between providers within the team in the rehabilitation centre (four items, *α* = 0.96) and cross-boundary continuity: between the rehabilitation centres and general practitioners in the municipality (four items, *α* = 0.95). The NCQ-N uses a 5-point Likert scale (one = strongly disagree, two = disagree, three = neutral, four = agree, five = strongly agree) with an option of “don’t know” (set as missing).

As adjustment variables we used variables; *age* and *sex* from the baseline survey. Variables; *marital status* and *education level* were register data provided by Statistics Norway and linked to the survey.

### Statistical analyses

Descriptive methods were used to describe sample characteristics. Missing data was handled with flexible multiple imputation method using chained predictive mean matching, creating 50 datasets [50]. Rubin’s rules were used for pooling the results [50].

Linear models were used to assess the association between RC and NCQ-N as independent variables and the WHODAS 2.0 domain and global scores and EQ-VAS score at 1-year as dependent variables. To express change in WHODAS 2.0 and EQ-VAS from baseline to follow-up, the model was also adjusted for WHODAS 2.0 and EQ-VAS baseline scores [51]. All models were adjusted for: sex, age (categorised as: 18–29, 30–39, 40–49, 50–59, 60–69 and > 70), marital status(categorised as: married, unmarried [not divorced], divorced), education level (categorised as: elementary school, high school and university/college) and health conditions, based on the Statistical Classification of Diseases and Related Health Problems Tenth Revision (ICD-10) referral diagnosis grouped as: neoplasms, nervous system diseases, musculoskeletal system diseases, circulatory system diseases, and others. Additionally, we made corresponding analyses including an interaction between diagnoses and the independent variables to assess possibly different slopes for the various ICD-10 referral diagnosis groups. All RC scales at patient level were clustered because of the team allocation. This has been taken into account by adding a random intercept for team allocation in the models including RC, turning them to Linear Mixed Effects models (LME).

The level of significance was set as 0.05. All statistical analyses were performed with IBM SPSS for Windows version 24 (IBM Corp. Armonk, NY) [52], and STATA 15 (STATA Corp., College Station, TX) [53]. The graphics were produced using Matlab 9.0 (The Mathworks Inc., Natrick, MA).

## Results

Patients reported a mean WHODAS 2.0 global score at 28.6 (standard deviation [SD] = 15.4) at baseline, which decreased to 24.1 (SD = 15.9) at 1-year follow-up, indicating reduced disability. Patients with neoplasms reported a larger reduction of disability, as measured by WHODAS 2.0 global score, compared to patients in other referral diagnosis groups included in this study (Table 2). The mean EQ-VAS score changed from 51.4 (SD = 18.8) at baseline to 58.2 (SD = 20.1) at 1-year follow-up, indicating improved self-rated health. Generally, patients reported largest reduction of disability for the WHODAS 2.0 domains: life activities, mobility and participation domains (Table 2). The neoplasms patient group shows a market reduction of disability in most WHODAS 2.0 domain scores and EQ-VAS score compared to other referral diagnosis groups included in this study (Supplementary Table 1).

**Table 2:** Distribution of the World Health Organisation Disability Assessment Schedule 2.0 and the EuroQol EQ-VAS among 701 patients at baseline and 1-year follow-up from specialised rehabilitation centres in Western Norway during the first half of 2015 and 2016

	Baseline	1-year follow-up	Change score
	Mean (SD)	Mean (SD)	Mean (95% CI)
WHODAS 2.0 domain score (all patients)			
Cognition	16.4 (18.0)	14.3 (16.4)	− 2.1 (− 3.24, − 0.96)
Mobility	32.5 (25.4)	26.3 (25.2)	− 6.2 (− 7.77, − 4.63)
Self-care	11.0 (17.2)	8.4 (15.9)	− 2.6 (− 3.84, − 1.36)
Getting along	23.9 (20.7)	22.3 (21.4)	− 1.6 (− 2.93, − 0.27)
Life activities	43.5 (28.1)	34.8 (27.5)	− 8.7 (− 10.62, − 6.78)
Participation	39.4 (20.4)	34.6 (21.7)	− 4.8 (− 6.10, − 3.50)
WHODAS 2.0 global score (all patients)	28.6 (15.4)	24.1 (15.9)	− 4.5 (− 5.42, − 3.58)
Neoplasms	30.3 (15.4)	20.1 (14.8)	− 10.2 (− 14.83, − 5.57)
Diseases in nervous systems	30.0 (14.2)	26.4 (14.0)	− 3.6 (− 6.08, − 1.18)
Diseases in musculoskeletal systems	26.6 (15.3)	22.2 (15.9)	− 4.4 (− 5.57, − 3.13)
Diseases in circulatory systems	32.6 (15.7)	28.4 (16.6)	− 4.2 (− 7.39, − 1.03)
Others	30.6 (15.0)	27.1 (16.3)	− 3.5 (− 5.48, − 1.52)
EQ-VAS (all patients)	51.4 (18.8)	58.2 (20.1)	7.2 (5.85, 8.55)
Neoplasms	51.7 (19.7)	63.4 (21.9)	10.2 (3.17, 17.17)
Diseases in nervous systems	46.1 (18.9)	56.3 (18.3)	9.7 (5.92, 13.52)
Diseases in musculoskeletal systems	53.0 (18.7)	59.9 (19.8)	7.0 (5.29, 8.77)
Diseases in circulatory systems	47.4 (17.0)	55.2 (16.9)	8.0 (3.15, 12.83)
Others	50.6 (19.1)	54.6 (21.0)	4.6 (1.61, 7.53)

WHODAS 2.0, World Health Organization Disability Assessment Schedule version 2.0; EQ-VAS, EuroQol EQ-VAS; SD: standard deviation; 95% CI, 95% confidence interval; 1: WHODAS 2.0 domain and global score range from: 0 = no disability to 100 = full disability); 2: EQ-VAS range from, 0 = worst imaginable health state to 100 = best imaginable health state

The mean interprofessional team RC communication score for the patient group was 3.9 (SD = 0.31), and the mean team RC relationship score for the patient group was 4.1 (SD = 0.28) (Table 3). NCQ-N among patients ranged from 2.9 (SD = 0.91) for personal continuity, where respondents reported that the most important health care professional in the team “shows commitment”, to the highest mean score for team continuity within somatic rehabilitation centres of 3.7 (SD = 0.84) (Table 3).

**Table 3:** Relational coordination and Nijmegen Continuity Questionnaire-N subscale scores in the study population (*N* = 701)

	Mean (SD)
Relational coordination^a^	
RC communication	3.9 (0.31)
RC relationship	4.1 (0.28)
Nijmegen Continuity Questionnaire-Norwegian version	
NCQ-N personal continuity (“knows me”)	3.0 (0.83)
NCQ-N personal continuity (“shows commitment”)	2.9 (0.91)
NCQ-N team continuity (within somatic rehabilitation)	3.7 (0.84)
NCQ-N cross-boundary continuity (between rehabilitation centres and GP in municipality)	3.0 (0.92)

*RC* relational coordination,* NCQ-N* Nijmegen continuity questionnaire-Norwegian version,* GP* general practitioner,* SD* standard deviation

^a^All patients were connected to their respective treating team in the rehabilitation centre during their stay

No associations were found between RC and NCQ-N subscale with changes in WHODAS 2.0 global score (Table 4). There were associations between NCQ-N team continuity and change in WHODAS 2.0 cognition; − 1.54 (SD = 18.3, *p* = 0.027), NCQ-N team continuity and WHODAS 2.0 participation; − 2.09 (SD = 21.2, *p* = 0.009) and NCQ-N cross-boundary continuity and WHODAS 2.0 life activities; − 2.20 (SD = 29.7, *p* = 0.050); however, no associations were found between RC and changes in WHODAS 2.0 domain scores (Table 4).

**Table 4:** Associations of relational coordination in interprofessional teams and patient-rated continuity of care subscale scores with the changes in World Health Organisation Disability Assessment Schedule 2.0 global score (*N* = 701)

	WHODAS 2.0 domain and global score		
	Adjusted^a^		
	*b*	95% CI	*p* value
RC communication			
Cognition	− 2.36	− 6.12, 1.40	0.218
Mobility	− 0.75	− 8.91, 7.41	0.857
Self-care	− 0.91	− 5.51, 3.70	0.699
Getting along	− 1.93	− 6.80, 2.95	0.438
Life activities	− 2.25	− 10.64, 6.14	0.600
Participation	− 1.32	− 7.17, 4.53	0.658
*Global score*	− *1.04*	− *5.84, 3.75*	*0.670*
RC relationship			
Cognition	− 2.17	− 6.04, 1.71	0.274
Mobility	3.19	− 5.72, 12.10	0.482
Self-care	0.02	− 5.20, 5.23	0.995
Getting along	− 0.78	− 5.65, 4.10	0.755
Life activities	− 1.39	− 10.61, 7.81	0.766
Participation	0.59	− 6.08, 7.26	0.861
*Global score*	*0.86*	− *4.55, 6.27*	*0.755*
NCQ-N personal1			
Cognition	0.19	− 1.12, 1,50	0.777
Mobility	0.15	− 1.77, 2.08	0.877
Self-care	0.27	− 1.07, 1.62	0.688
Getting along	0.10	− 1.44, 1.64	0.897
Life activities	− 0.62	− 2.75, 1.50	0.566
Participation	− 0.74	− 2.28, 0.80	0.347
*Global score*	− *0.26*	− *1.37, 0.86*	*0.653*
NCQ-N personal2			
Cognition	− 0.01	− 1.19, 1.18	0.990
Mobility	− 0.76	− 2.50, 0.98	0.390
Self-care	0.15	− 1.04, 1.34	0.802
Getting along	− 0.45	− 1.87, 0.98	0.537
Life activities	− 0.81	− 2.79, 1.16	0.419
Participation	− 1.08	− 2.48, 0.32	0.132
*Global score*	− *0.58*	− *1.60, 0.43*	*0.260*
NCQ-N team			
Cognition	− 1.54	− 2.90, − 0.18	0.027
Mobility	− 0.79	− 2.64, 1.06	0.403
Self-care	− 0.30	− 1.73, 1.13	0.679
Getting along	− 1.59	− 3.26, 0.08	0.062
Life activities	− 0.40	− 2.66, 1.86	0.727
Participation	− 2.09	− 3.66, − 0.53	0.009
*Global score*	− *1.03*	− *2.19, 0.13*	*0.082*
NCQ-N cross-boundary			
Cognition	− 0.19	− 1.51, 1.13	0.775
Mobility	− 1.06	− 2.94, 0.82	0.270
Self-care	− 0.01	− 1.34, 1.31	0.986
Getting along	− 0.49	− 2.00, 1.01	0.521
Life activities	− 2.20	− 4.39, − 0.00	0.050
Participation	− 1.26	− 2.84, 0.31	0.115
*Global score*	− *0.79*	− *1.97, 0.38*	*0.186*

*WHODAS 2.0* World Health Organization Disability Assessment Schedule version 2.0,* RC* relational coordination subscale score, *NCQ-N* Nijmegen continuity questionnaire- Norwegian version,* b* unstandardized estimated regression coefficient,* CI* confidence interval,* NCQ-N Personal1* NCQ-N personal continuity (“knows me”),* NCQ-N Personal 2* NCQ-N personal continuity (“shows commitment”),* NCQ-N Team* NCQ-N team continuity (within somatic rehabilitation),* NCQ-N Cross-boundary* NCQ-N cross-boundary continuity (between rehabilitation centres and general practitioner in municipality)

^a^Adjusted for: patients’ age group, sex, health conditions, education level, marital status and baseline dependent variable subscale score (WHODAS 2.0)

**Fig. 2:**
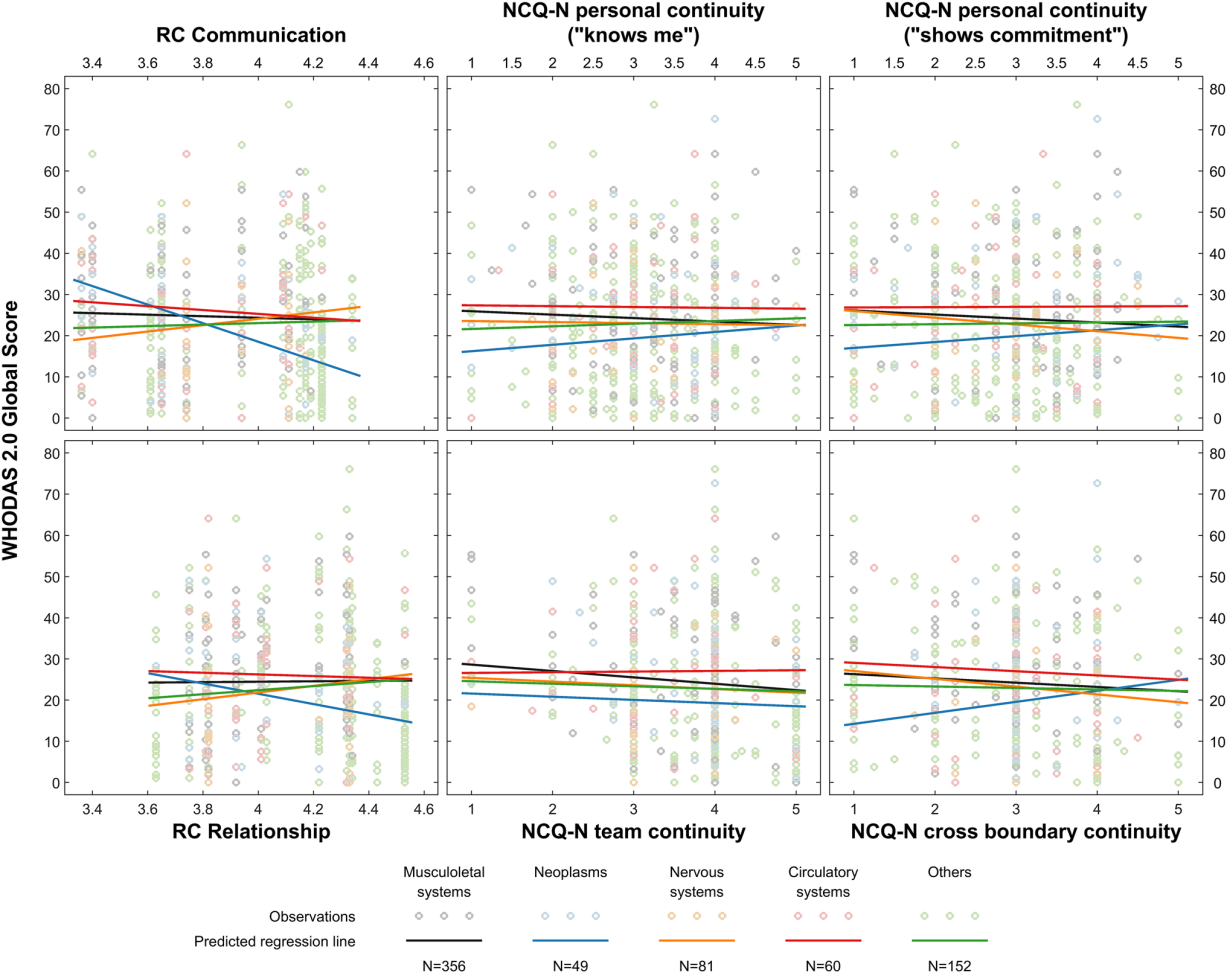
Associations of relational coordination subscale scores in interprofessional teams and patient-rated continuity of care subscale scores with the World Health Organisation Disability Assessment Schedule 2.0 global score with patients grouped by ICD-10 referral diagnoses (*N* = 701).* WHODAS 2.0* World Health Organization Disability Assessment Schedule version 2.0,* RC* relational coordination,* NCQ-N* Nijmegen continuity questionnaire-Norwegian version

Figure 2 presents analyses of associations between RC and NCQ-N subscale scores and changes in WHODAS 2.0 global scores for patient grouped by referral diagnosis. A higher RC communication score was associated with improved health for the neoplasms patient group (*b* = − 20.66, 95% CI = − 37.05, − 4.28, *p* = 0.013) (Supplementary Table 3). A similar (not significant) pattern can be seen between RC relationship and WHODAS 2.0 global scores for the neoplasms patient group. This study did not disclose associations between NCQ-N and changes in WHODAS 2.0 global score when analysing referral diagnosis groups separately. Supplementary Table 3 provides b coefficient, 95% CI and *p* values related to Fig. 2.

We found significant associations between all NCQ-N subscales and changes in the EQ-VAS (Table 5), while no associations were found between RC and changes in EQ-VAS.

**Table 5:** Associations of relational coordination subscale scores in interprofessional teams and patient-rated continuity of care subscale scores with the EuroQol EQ-VAS health state score (*N* = 701)

	EQ-VAS score		
	Adjusted^a^		
	*b*	95% CI	*p* value
RC communication	0.99	− 5.49, 7.46	0.764
RC relationship	0.27	− 6.90, 7.44	0.941
NCQ-N Personal1	2.50	0.94, 4.06	0.002
NCQ-N Personal2	2.28	0.81, 3.76	0.002
NCQ-N team	1.73	0.11, 3.35	0.037
NCQ-N cross-boundary	2.40	0.84, 3.96	0.003

*EQ-VAS* EuroQol EQ-VAS,* RC* relational coordination subscale score,* NCQ-N* Nijmegen continuity questionnaire-Norwegian version,* b* unstandardized estimated regression coefficient,* CI* confidence interval,* Personal1* NCQ-N personal continuity (“knows me”),* Personal 2* NCQ-N personal continuity (“shows commitment”),* Team* NCQ-N team continuity (within somatic rehabilitation),* Cross-boundary* NCQ-N cross-boundary continuity (between rehabilitation centres and general practitioner in municipality)

^a^Fully adjusted model is adjusted for: patients’ age group, sex, health conditions, education level, marital status and baseline dependent variable subscale score (EQ-VAS)

**Fig. 3:**
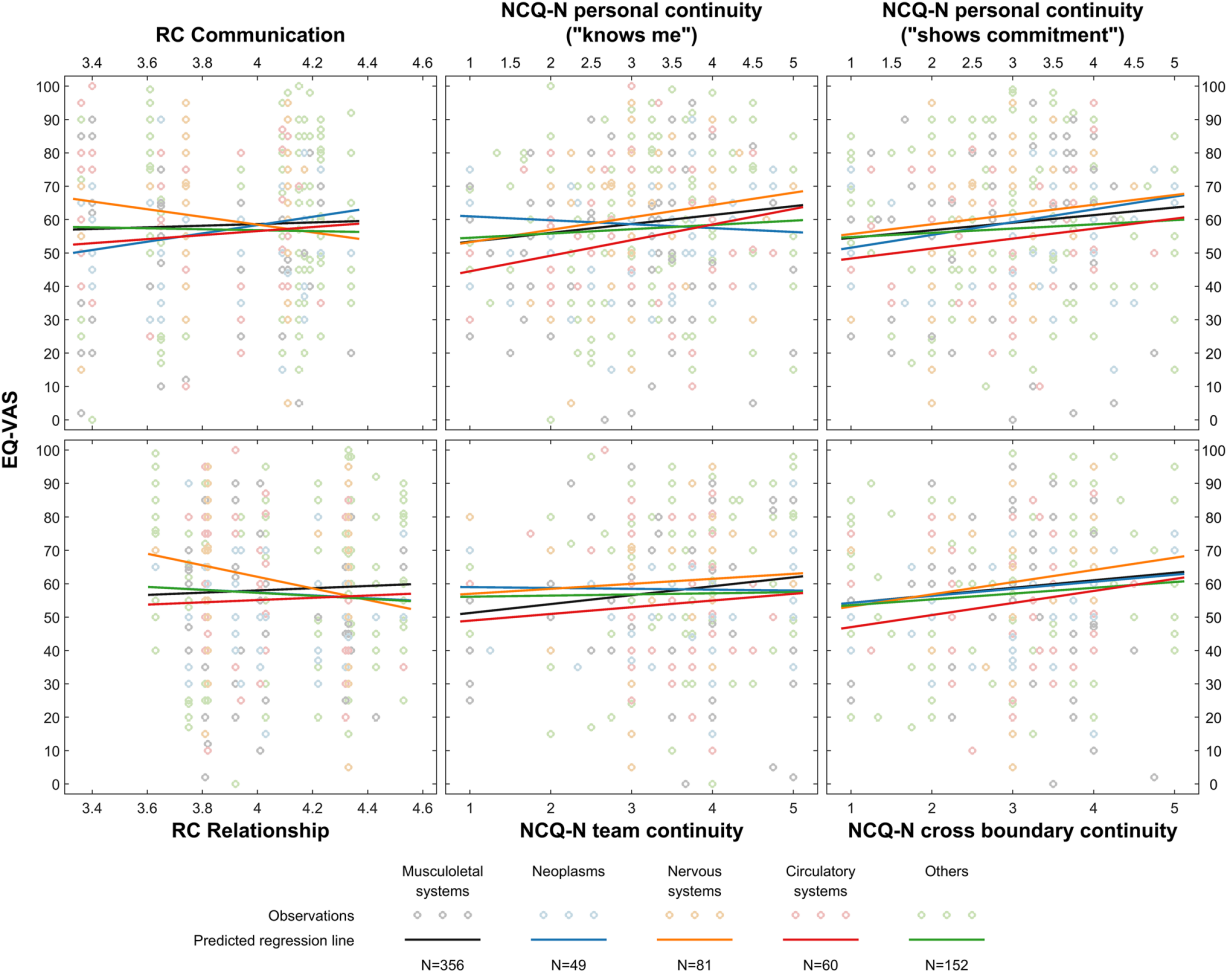
Associations of relational coordination subscale scores in interprofessional teams and patient-rated continuity of care subscale scores with the EuroQol EQ-VAS health state score with patients grouped by ICD-10 referral diagnoses (*N* = 701). *EQ-VAS* EuroQol EQ-VAS,* RC* relational coordination,* NCQ-N* Nijmegen continuity questionnaire-Norwegian version

Figure 3 presents analyses of associations between RC and NCQ-N subscale scores with changes in EQ-VAS scores for patients grouped by referral diagnosis. Patients referred with nervous system diseases reported a decrease in the EQ-VAS score when treated by teams with higher levels of RC relationship score (*b* = − 20.66, 95% CI = − 38.96, − 2.36, *p* = 0.027) (Supplementary Table 4), a similar (not significant) association was seen between RC communication score and EQ-VAS score in the same patient group. This study found that patients in all referral diagnosis groups reported improvement in health when experiencing continuity of care. Supplementary Table 4 provides b coefficient, 95% CI and *p* values related to Fig. 3.

## Discussion

To the best of our knowledge, this is the first study to investigate the associations between team functions in somatic rehabilitation centres and changes in health and disability among rehabilitation patients. An improvement of health was associated with better patient-reported continuity of care regarding rehabilitation care. However, continuity of care was not associated with reduced disability. Communication and relationship in teams, as reported by the professionals, were not associated with improvement in health or decreased disability, looking at the total sample. However, neoplasms patient group improved their health more compared to other diagnosis groups included in this study.

Previous studies have reported that continuity of care is associated with reduced length of stay in hospital, reduced readmission rates, reduced cost, and increased patient satisfaction as outcomes [12, 16, 54, 55]. However, relatively few studies have investigated the associations between continuity of care and patient-rated health outcomes. The present study expands knowledge in this field, revealing a significant association between both personal continuity and team continuity in the rehabilitation team on one hand, and improved health after rehabilitation stay on the other. However, we found no association between continuity of care and changes in the level of disability. These findings indicate a need for more research to verify the impact of continuity of care on patients’ outcomes, preferably with more direct measures of health and functioning.

The importance of teams working towards shared goals using a shared approach in health care settings has a well-established theoretical and empirical basis, and found to positively influence the quality and continuity of patient care [56–59]. One would therefore assume that a higher score on RC in rehabilitation teams would positively affect patients’ health and disability. This present study found that the neoplasms patient group reported a greater improvement in function compared to the other patient groups included. This is in line with previous research that found communication in interprofessional teams to positively impact patient outcomes of cancer care [60]. In our study, this patient group showed the most marked improvement in functioning during the study period. One explanation for this finding could be that this patient group represents a selection of patients who had recently undergone treatment prior to commencing a rehabilitation stay and therefore could be more inclined to be in a phase of recovery where the intervention by rehabilitation teams is especially useful. Patients with nervous system diseases treated by teams with better team functions as measured by RC reported a decrease in health, as measured by EQ-VAS. These patients often have progressive diseases, and one explanation for this finding could be that patients with most serious condition are of greater need for team functions due to a more severe decline in health over time, compared to other diagnosis groups included in this study.

In a previous study, we found that RC communication and relationships in teams were inversely associated with personal continuity as reported by the patient after rehabilitation [18]. Thus, patients treated by a well-functioning team, as defined by RC, were unlikely to specifically have a close relationship with the most important professional during their rehabilitation stay. This is contradictory to previous research reporting that team-based models was associated with increased social participation among stroke patients [61]. However, in these models the patient had a defined coordinator, responsible for systematic follow-up after a rehabilitation process. The present study found an association between personal continuity and improvement in health, as measured by EQ-VAS. This effect of personal continuity is well documented in other care settings [16, 17, 21, 62]. Further, in accordance with previous research [12, 55], this current study found continuity of care to positively influence patient-rated changes in health after a rehabilitation stay. One explanation for these findings could be that continuity of care as defined and experienced by patients may differ from continuity of care as defined by health care professionals. The lack of personal continuity might be a limitation of team-based care and should be taken into account when organising rehabilitation care.

Since the present study focused on the health outcomes after rehabilitation, we also looked at cross-boundary continuity between rehabilitation centres and primary health care. Patients may have received health care services in the municipality to follow up interventions received at the rehabilitation centre. Interprofessional rehabilitation teams communicate with other health care professionals across settings, and the current results revealed that better cross-boundary continuity in the NCQ-N was associated with improved health outcomes. This finding is in line with previous studies reporting that a lack of continuity across settings was associated with an increased risk of inactivity, falls and readmission among stroke patients [63]. Further, previous studies have shown that continuity of care after hospital discharge was associated with a reduced risk of death and readmission to hospital [54, 55].

### Study strengths and limitations

An important strength of the current study was the longitudinal design and the comprehensive study population with a broad range of health conditions. In addition, this study included patients who were accepted for somatic rehabilitation in all rehabilitation centres in a defined geographical area (Western Norway), combined with data collection from employees working in interprofessional rehabilitation teams. However, a major limitation was the low response rate at baseline (34%) and at 1-year follow-up (25% of the patients recruited at baseline), which may have resulted in selection bias and problems regarding representability. A further limitation was loss of participants at 1-year follow-up. As non-responders at follow-up seemed to be younger and more often male compared to the responders, an attrition bias could have affected findings. Changes in health at 1-year follow-up could be smaller due to including a sample with a higher mean age and increased number of women.

Strength of the current study was the use of validated generic survey instruments, which enabled us to study a heterogeneous rehabilitation patient cohort. The instruments have shown satisfactory psychometric properties in terms of factor structure and reliability, and the WHODAS 2.0 had satisfactory test–retest reliability [22]. The instruments used were valid and reliable for capturing patient-rated health and disability. However, several limitations regarding the included instruments should be considered. The NCQ-N included the response option “don’t know”, which, in this study, was set as “missing”. This resulted in a relatively large number of missing data points. However, using a flexible multiple imputation method for handling missing data reduced the potential effects of bias due to a large number of missing data points in the NCQ-N responses. The low variance in RC between teams may make it difficult to disclose eventual associations between RC in teams and patient-rated outcomes, and our findings should be interpreted with this precaution. The results of the analyses regarding referral diagnosis groups should be interpreted cautiously as some patient groups were relatively small and our findings may therefore not be generalizable to these groups at large. A further potential limitation is that patients in the present study reported mild to moderate disability level according to the WHODAS 2.0 global scale, which may limit the generalisability of the current results to populations with more severe disability.

## Conclusion

The current study revealed that better personal, team and cross-boundary continuity of rehabilitation care was associated with improved health after rehabilitation. Measures of patient-rated personal, team and cross-boundary continuity may be a promising indicator of the quality of rehabilitation care. However, our findings did not reveal any associations between RC in interprofessional teams and self-rated health or disability among rehabilitation patients. More research is needed to understand the effects of team functioning in interprofessional rehabilitation teams on patient health outcomes.

## Electronic supplementary material

Below is the link to the electronic supplementary material.

Supplementary material 1 (DOCX 41 kb)

## Data Availability

The raw data are property of the research unit in the Bergen Health Authority (Helse Bergen) and are available on a reasonable request.

## Abbreviations

RC: Relational coordination
NCQ: Nijmegen Continuity Questionnaire
NCQ-N: Norwegian version of the Nijmegen Continuity Questionnaire
GP: General practitioner
WHODAS 2.0: World Health Organization Disability Assessment Schedule 2.0
EQ-5D-5L: EuroQol-5 dimension descriptive system
EQ-VAS: EuroQol EQ-VAS
b: Unstandardized estimated regression coefficient
SD: Standard deviation
CI: Confidence interval
