# Supplementary material for: The effect of team collaboration and continuity of care on health and disability among rehabilitation patients: a longitudinal survey-based study from western Norway
Source: Qual Life Res. 2019 May 29;28(10):2773–85. doi: 10.1007/s11136-019-02216-7 (PMC6761089; doi:10.1007/s11136-019-02216-7)
Supplement: Supplementary file 1 — Supplementary material 1 (DOCX 41 kb) [file 11136_2019_2216_MOESM1_ESM.docx]

**Supplementary table 1.** Distribution of the World Health Organisation Disability Assessment Schedule 2.0 domain and global scores and the EuroQol EQ-VAS among 701 patients at baseline and 1-year follow-up from secondary rehabilitation centres in Western Norway grouped by ICD-10 referral diagnoses.

|  |  |  |  | **Baseline** |  | **1-year follow-up** |  | **Change score** |
| --- | --- | --- | --- | --- | --- | --- | --- | --- |
|  |  |  |  | **Mean (SD)** |  | **Mean (SD)** |  | **Mean (95% CI)** |
|  |  |  |  |  |  |  |  |  |
| **WHODAS 2.0 domain score** | | |  |  |  |  |  |  |
|  | **Cognition** | |  | **16.4 (18.0)** |  | **14.3 (16.4)** |  | **–2.1 (–3.24, –0.96)** |
|  |  | Neoplasms |  | 15.4 (19.5) |  | 10.4 (13.6) |  | –5.0 (–9.81, –0.21) |
|  |  | Diseases in nervous system |  | 18.3 (18.2) |  | 16.3 (16.3) |  | –2.0 (–5.40, 1.42) |
|  |  | Diseases in musculoskeletal systems |  | 15.8 (17.4) |  | 13.1 (16.6) |  | –2.8 (–4.24, –1.26) |
|  |  | Diseases in circulatory systems |  | 18.3 (21.1) |  | 15.3 (16.6) |  | –3.0 (–7.33, 1.25) |
|  |  | Others† |  | 16.3 (17.5) |  | 16.9 (17.1) |  | 0.6 (–1.99, 3.15) |
|  | **Mobility** | |  | **32.5 (25.4)** |  | **26.3 (25.2)** |  | **–6.2 (–7.77, –4.63)** |
|  |  | Neoplasms |  | 45.8 (28.3) |  | 28.0 (24.0) |  | –17.8 (–26.28, –9.34) |
|  |  | Diseases in nervous system |  | 35.4 (23.0) |  | 32.3 (25.7) |  | –3.1 (–11.27, 5.07) |
|  |  | Diseases in musculoskeletal systems |  | 28.5 (24.7) |  | 23.0 (23.8) |  | –5.5 (–7.54, –3.50) |
|  |  | Diseases in circulatory systems |  | 33.9 (26.3) |  | 30.0 (27.4) |  | –3.9 (–9.02, 1.24) |
|  |  | Others† |  | 35.4 (25.3) |  | 29.0 (26.5) |  | –6.4 (–9.92, –2.82) |
|  | **Self-care** | |  | **11.0 (17.2)** |  | **8.4 (15.9)** |  | **–2.6 (–3.84, –1.36)** |
|  |  | Neoplasms |  | 18.7 (21.4) |  | 7.5 (13.0) |  | –11.3 (–17.10, –5.41) |
|  |  | Diseases in nervous system |  | 13.0 (17.8) |  | 10.2 (16.7) |  | –2.7 (–5.82, 0.34) |
|  |  | Diseases in musculoskeletal systems |  | 8.6 (15.1) |  | 7.0 (15.3) |  | –1.6 (–3.36, 0.12) |
|  |  | Diseases in circulatory systems |  | 11.3 (18.4) |  | 10.0 (17.7) |  | –1.3 (–5.43, 2.77) |
|  |  | Others† |  | 12.8 (18.1) |  | 10.5 (16.2) |  | –2.3 (–4.91, 0.31) |
|  | **Getting along** | |  | **23.9 (20.7)** |  | **22.3 (21.4)** |  | –**1.6 (–2.93, –0.27)** |
|  |  | Neoplasms |  | 19.3 (15.7) |  | 18.2 (17.6) |  | –1.1 (–5.41, 3.25) |
|  |  | Diseases in nervous system |  | 21.7 (17.8) |  | 21.3 (19.9) |  | –0.4 (–3.78, 3.04) |
|  |  | Diseases in musculoskeletal systems |  | 23.2 (20.6) |  | 21.7 (20.8) |  | –1.5 (–3.31, 0.33) |
|  |  | Diseases in circulatory systems |  | 28.1 (25.6) |  | 27.7 (27.0) |  | –0.4 (–6.04, 5.16) |
|  |  | Others† |  | 26.4 (21.3) |  | 23.2 (21.3) |  | –3.2 (–6.34, –0.16) |
|  | **Life activities** | |  | **43.5 (28.1)** |  | **34.8 (27.5)** |  | –**8.7 (–10.62, –6.78)** |
|  |  | Neoplasms |  | 47.8 (30.5) |  | 28.7 (23.7) |  | –19.0 (–29.30, –9.14) |
|  |  | Diseases in nervous system |  | 47.0 (26.1) |  | 37.8 (25.6) |  | –9.1 (–15.29, –2.99) |
|  |  | Diseases in musculoskeletal systems |  | 39.9 (27.4) |  | 31.7 (27.2) |  | –8.3 (–10.84, –5.74) |
|  |  | Diseases in circulatory systems |  | 49.9 (29.1) |  | 41.9 (29.0) |  | –8.0 (–13.55, –2.37) |
|  |  | Others† |  | 46.3 (29.0) |  | 40.1 (28.2) |  | –6.2 (–10.20, –2.24) |
|  | **Participation** | |  | **39.4 (20.4)** |  | **34.6 (21.7)** |  | –**4.8 (–6.10, –3.50)** |
|  |  | Neoplasms |  | 39.5 (22.1) |  | 28.4 (19.7) |  | –11.1 (–17.10, –5.02) |
|  |  | Diseases in nervous system |  | 39.4 (18.0) |  | 36.6 (19.4) |  | –2.9 (–6.06, 0.30) |
|  |  | Diseases in musculoskeletal systems |  | 37.4 (20.6) |  | 32.5 (20.9) |  | –4.9 (–6.57, –3.27) |
|  |  | Diseases in circulatory systems |  | 44.7 (18.9) |  | 41.1 (20.6) |  | –3.7 (–7.89, 0.53) |
|  |  | Others† |  | 41.9 (20.8) |  | 38.2 (23.8) |  | –3.8 (–6.93, –0.61) |
|  | **WHODAS 2.0 global score** | |  | **28.6 (15.4)** |  | **24.1 (15.9)** |  | **–4.5 (–5.42, –3.58)** |
|  |  | Neoplasms |  | 30.3 (15.4) |  | 20.1 (14.8) |  | –10.2 (–14.83, –5.57) |
|  |  | Diseases in nervous system |  | 30.0 (14.2) |  | 26.4 (14.0) |  | –3.6 (–6.08, –1.18) |
|  |  | Diseases in musculoskeletal systems |  | 26.6 (15.3) |  | 22.2 (15.9) |  | –4.4 (–5.57, –3.13) |
|  |  | Diseases in circulatory systems |  | 32.6 (15.7) |  | 28.4 (16.6) |  | –4.2 (–7.39, –1.03) |
|  |  | Others† |  | 30.6 (15.0) |  | 27.1 (16.3) |  | –3.5 (–5.48, –1.52) |
|  | **EQ-VAS** | |  | **51.4 (18.8)** |  | **58.2 (20.1)** |  | **7.2 (5.85, 8.55)** |
|  |  | Neoplasms |  | 51.7 (19.7) |  | 63.4 (21.9) |  | 10.2 (3.17, 17.17) |
|  |  | Diseases in nervous system |  | 46.1 (18.9) |  | 56.3 (18.3) |  | 9.7 (5.92, 13.52) |
|  |  | Diseases in musculoskeletal systems |  | 53.0 (18.7) |  | 59.9 (19.8) |  | 7.0 (5.29, 8.77) |
|  |  | Diseases in circulatory systems |  | 47.4 (17.0) |  | 55.2 (16.9) |  | 8.0 (3.15, 12.83) |
|  |  | Others† |  | 50.6 (19.1) |  | 54.6 (21.0) |  | 4.6 (1.61, 7.53) |

Abbreviations: WHODAS 2.0: World Health Organization Disability Assessment Schedule version 2.0; EQ-VAS: EuroQol EQ-VAS; SD: standard deviation; 95% CI: 95% confidence interval; 1: WHODAS 2.0 domain and global score range from: 0=no disability to 100=full disability); 2: EQ-VAS range from: 0=worst imaginable health state to 100=best imaginable health state

† Other health conditions included the following: endocrine, nutritional and metabolic diseases (n=36); respiratory diseases (n=35); diseases of the skin and subcutaneous tissue (23); injuries and external causes (n=18); factors influencing self-rated health and contact with services (n=7); mental and behavioural disorders (n=12); symptoms, sign and abnormal clinical and laboratory findings, not elsewhere classified (n=4); codes for special purposes (n=6); diseases of the digestive system (n= 5); diseases of the blood and blood-forming organs, and certain disorders involving the immune mechanism (n=1); diseases of the ear and the mastoid process (n=1); diseases of the genitourinary system (n=1); congenital malfunctions, and chromosomal abnormalities (n=1); and certain infectious and parasitic diseases (n=2).

**Supplementary table 2.** Associations of Relational Coordination subscale scores in interprofessional teams and continuity of care subscale scores with changes in the World Health Organisation Disability Assessment Schedule 2.0 global score with patients grouped by ICD-10 referral diagnoses (N=701).

|  |  |  |  | **WHODAS 2.0 Global score** | | |
| --- | --- | --- | --- | --- | --- | --- |
|  |  |  |  | **Adjusted*** | | |
|  |  |  |  | **b** | **95% CI** | **p-value** |
| **RC Communication, main effect^±^** | | |  | –1.04 | – 5.84, 3.75 | 0.670 |
|  | **Model with interaction**^£^**:** | |  |  |  |  |
|  | **Reference diagnosis (musculoskeletal)** | |  | –1.87 | –7.60, 3.87 | 0.524 |
|  |  | Neoplasms |  | –20.66 | –37.05, -4.28 | 0.013 |
|  |  | Diseases in the nervous system |  | 9.61 | –0.24, 19.46 | 0.056 |
|  |  | Diseases in the circulatory systems |  | –2.82 | –14.60, 8.96 | 0.639 |
|  |  | Others† |  | 3.67 | –4.41, 11.76 | 0.373 |
| **RC Relationship, main effect^±^** | | |  | 0.86 | –4.55, 6.27 | 0.755 |
|  | **Model with interaction**^£^**:** | |  |  |  |  |
|  | **Reference diagnosis (musculoskeletal)** | |  | 0.52 | –5.65, 6.69 | 0.869 |
|  |  | Neoplasms |  | –13.04 | –27.47, 1.39 | 0.076 |
|  |  | Diseases in the nervous system |  | 7.57 | –4.98, 1.39 | 0.237 |
|  |  | Diseases in the circulatory systems |  | –2.53 | –17.04, 11.97 | 0.732 |
|  |  | Others† |  | 4.40 | –5.19, 13.98 | 0.369 |
| **NCQ-N Personal1, main effect^±^** | | |  | –0.26 | –1.37, 0.86 | 0.653 |
|  | **Model with interaction**^£^**:** | |  |  |  |  |
|  | **Reference diagnosis (musculoskeletal)** | |  | –0.83 | –2.35, 0.69 | 0.283 |
|  |  | Neoplasms |  | 2.39 | –2.38, 7.16 | 0.326 |
|  |  | Diseases in the nervous system |  | 0.59 | –2.95, 4.12 | 0.745 |
|  |  | Diseases in the circulatory systems |  | 0.63 | –3.15, 4.41 | 0.744 |
|  |  | Others† |  | 1.48 | –1.37, 4.32 | 0.309 |
| **NCQ-N Personal2, main effect^±^** | | |  | –0.58 | –1.60, 0.43 | 0.260 |
|  | **Model with interaction**^£^**:** | |  |  |  |  |
|  | **Reference diagnosis (musculoskeletal)** | |  | –0.97 | –2.36, 0.41 | 0.167 |
|  |  | Neoplasms |  | 2.41 | –3.08, 7.89 | 0.389 |
|  |  | Diseases in the nervous system |  | –0.66 | –3.77, 2.45 | 0.679 |
|  |  | Diseases in the circulatory systems |  | 1.05 | –2.35, 4.45 | 0.543 |
|  |  | Others† |  | 1.18 | –1.38, 3.74 | 0.366 |
| **NCQ-N Team, main effect^±^** | | |  | –1.03 | –2.19, 0.13 | 0.082 |
|  | **Model with interaction**^£^**:** | |  |  |  |  |
|  | **Reference diagnosis (musculoskeletal)** | |  | –1.55 | –3.16, 0.06 | 0.060 |
|  |  | Neoplasms |  | 0.78 | –4.32, 5.88 | 0.763 |
|  |  | Diseases in the nervous system |  | 0.65 | –3.13, 4.43 | 0.736 |
|  |  | Diseases in the circulatory systems |  | 1.71 | –2.33, 5.76 | 0.406 |
|  |  | Others† |  | 0.94 | –1.80, 3.67 | 0.503 |
| **NCQ-N Cross- boundary, main effect^±^** | | |  | –0.79 | –1.97, 0.38 | 0.186 |
|  | **Model with interaction**^£^**:** | |  |  |  |  |
|  | **Reference diagnosis (musculoskeletal)** | |  | -1.04 | -2.44, 0.36 | 0.145 |
|  |  | Neoplasms |  | 3.71 | –1.62, 9.05 | 0.172 |
|  |  | Diseases in the nervous system |  | –0.86 | –4.35, 2.64 | 0.631 |
|  |  | Diseases in the circulatory systems |  | 0.02 | –3.84, 3.89 | 0.991 |
|  |  | Others† |  | 0.68 | –1.93, 3.29 | 0.610 |

Abbreviations: WHODAS 2.0: World Health Organization Disability Assessment Schedule version 2.0; RC: Relational coordination sub-scale score; NCQ-N: Nijmegen continuity questionnaire- Norwegian version; ICD-10: International Statistical Classification of Diseases and Related Health Problems, Tenth Revision; NCQ-N Personal1: NCQ-N personal continuity (“knows me”); NCQ-N Personal 2: NCQ-N personal continuity (“shows commitment”); NCQ-N Team: NCQ-N team continuity (within somatic rehabilitation); NCQ-N Cross-boundary: NCQ-N cross-boundary continuity (between rehabilitation centres and general practitioner in municipality)

† Other health conditions included the following: endocrine, nutritional and metabolic diseases (n=36); respiratory diseases (n=35); diseases of the skin and subcutaneous tissue (23); injuries and external causes (n=18); factors influencing self-rated health and contact with services (n=7); mental and behavioural disorders (n=12); symptoms, sign and abnormal clinical and laboratory findings, not elsewhere classified (n=4); codes for special purposes (n=6); diseases of the digestive system (n= 5); diseases of the blood and blood-forming organs, and certain disorders involving the immune mechanism (n=1); diseases of the ear and the mastoid process (n=1); diseases of the genitourinary system (n=1); congenital malfunctions, and chromosomal abnormalities (n=1); and certain infectious and parasitic diseases (n=2).

* Adjusted for: patients’ age group, sex, health conditions, education level, marital status and baseline dependent variable subscale score (WHODAS 2.0).

**±**Main effect are results from the linear regression models presented in table 5 in the paper.

£ Interaction between RC/NCQ-N and ICD-10 referral diagnosis groups

**Supplementary table 3.** Associations of Relational Coordination subscale scores in interprofessional teams and continuity of care subscale scores with changes in the European Quality of Life visual analogue scale health state score with patients grouped by ICD-10 referral diagnoses (N=701).

|  |  |  |  | **EQ-VAS** | | |
| --- | --- | --- | --- | --- | --- | --- |
|  |  |  |  | **Adjusted*** | | |
|  |  |  |  | **b** | **95% CI** | **p-value** |
| **RC Communication, main effect^±^** | | |  | 0.99 | –5.49, 7.46 | 0.764 |
|  | **Main effect (Mean for diagnoses)** | |  |  |  |  |
|  | **Model with interaction^£^:** | |  | 2.44 | –5.50, 10.38 | 0.547 |
|  |  | Neoplasms |  | 10.00 | –12.65, 32.65 | 0.387 |
|  |  | Diseases in the nervous system |  | –13.99 | –28.40, 0.42 | 0.057 |
|  |  | Diseases in the circulatory systems |  | 3.60 | –11.64, 18.84 | 0.643 |
|  |  | Others† |  | –3.86 | –15.56, 7.85 | 0.518 |
| **RC Relationship, main effect^±^** | | |  | 0.27 | –6.90, 7.44 | 0.941 |
|  | **Model with interaction^£^:** | |  |  |  |  |
|  | **Reference diagnosis (musculoskeletal)** | |  | 3.35 | –4.90, 11.60 | 0.426 |
|  |  | Neoplasms |  | –7.59 | –28.61, 13.42 | 0.479 |
|  |  | Diseases in the nervous system |  | –20.66 | –38.96, -2.36 | 0.027 |
|  |  | Diseases in the circulatory systems |  | 0.05 | –18.95, 19.04 | 0.996 |
|  |  | Others† |  | –7.80 | –22.00, 6.40 | 0.281 |
| **NCQ-N Personal1, main effect^±^** | | |  | 2.50 | 0.94, 4.06 | 0.002 |
|  | **Model with interaction^£^:** | |  |  |  |  |
|  | **Reference diagnosis (musculoskeletal)** | |  | 2.67 | 0.49, 4.84 | 0.016 |
|  |  | Neoplasms |  | –3.84 | –10.81, 3.12 | 0.280 |
|  |  | Diseases in the nervous system |  | 1.04 | –3.86, 5.94 | 0.677 |
|  |  | Diseases in the circulatory systems |  | 2.00 | –3.18, 7.19 | 0.449 |
|  |  | Others† |  | –1.38 | –5.41, 2.65 | 0.501 |
| **NCQ-N Personal2, main effect^±^** | | |  | 2.28 | 0.81, 3.76 | 0.002 |
|  | **Model with interaction^£^:** | |  |  |  |  |
|  | **Reference diagnosis (musculoskeletal)** | |  | 2.27 | 0.26, 4.28 | 0.027 |
|  |  | Neoplasms |  | 1.56 | –5.80, 8.92 | 0.678 |
|  |  | Diseases in the nervous system |  | 0.65 | –3.94, 5.25 | 0.780 |
|  |  | Diseases in the circulatory systems |  | 0.72 | –4.00, 5.43 | 0.766 |
|  |  | Others† |  | –1.00 | –4.69, 2.70 | 0.596 |
| **NCQ-N Team, main effect^±^** | | |  | 1.73 | 0.11, 3.35 | 0.037 |
|  | **Model with interaction^£^:** | |  |  |  |  |
|  | **Reference diagnosis (musculoskeletal)** | |  | 2.67 | 0.36, 4.98 | 0.023 |
|  |  | Neoplasms |  | –2.93 | –9.65, 3.80 | 0.393 |
|  |  | Diseases in the nervous system |  | –1.17 | –6.64, 4.30 | 0.675 |
|  |  | Diseases in the circulatory systems |  | –0.66 | –6.12, 4.80 | 0.813 |
|  |  | Others† |  | –2.33 | –6.34, 1.68 | 0.255 |
| **NCQ-N Cross- boundary, main effect^±^** | | |  | 2.40 | 0.84, 3.96 | 0.003 |
|  | **Model with interaction^£^:** | |  |  |  |  |
|  | **Reference diagnosis (musculoskeletal)** | |  | 2.26 | 0.31, 4.20 | 0.023 |
|  |  | Neoplasms |  | –0.14 | –7.72, 7.43 | 0.970 |
|  |  | Diseases in the nervous system |  | 1.41 | –3.75, 6.57 | 0.593 |
|  |  | Diseases in the circulatory systems |  | 1.36 | –4.29, 7.00 | 0.637 |
|  |  | Others† |  | –0.52 | –4.42, 3.38 | 0.794 |

Abbreviations: EQ-VAS: EuroQol EQ-VAS; RC: Relational coordination sub-scale score; NCQ-N: Nijmegen continuity questionnaire- Norwegian version; b: unstandardized estimated regression coefficient; CI: confidence interval; NCQ-N Personal1: NCQ-N personal continuity (“knows me”); NCQ-N Personal 2: NCQ-N personal continuity (“shows commitment”); NCQ-N Team: NCQ-N team continuity (within somatic rehabilitation); NCQ-N Cross-boundary: NCQ-N cross-boundary continuity (between rehabilitation centres and general practitioner in municipality)

† Other health conditions included the following: endocrine, nutritional and metabolic diseases (n=36); respiratory diseases (n=35); diseases of the skin and subcutaneous tissue (23); injuries and external causes (n=18); factors influencing self-rated health and contact with services (n=7); mental and behavioural disorders (n=12); symptoms, sign and abnormal clinical and laboratory findings, not elsewhere classified (n=4); codes for special purposes (n=6); diseases of the digestive system (n= 5); diseases of the blood and blood-forming organs, and certain disorders involving the immune mechanism (n=1); diseases of the ear and the mastoid process (n=1); diseases of the genitourinary system (n=1); congenital malfunctions, and chromosomal abnormalities (n=1); and certain infectious and parasitic diseases (n=2).

* Adjusted for: patients’ age group, sex, health conditions, education level, marital status and baseline dependent variable subscale score (EQ-VAS).

**±**Main effect are results from the linear regression models presented in table 5 in the paper.

£ Interaction between RC/NCQ-N and ICD-10 referral diagnosis groups
